# Epilepsy in older adults: a comparison of early-onset persistent and late-onset epilepsy

**DOI:** 10.1007/s00415-026-13916-9

**Published:** 2026-06-09

**Authors:** Nicoletta Dörr, Thea Hüsing, Hanna Kleespies, Martin Holtkamp, Jakob I. Doerrfuss

**Affiliations:** 1 Department of Neurology with Experimental Neurology, Charité – Universitätsmedizin Berlin, corporate member of Freie Universität Berlin and Humboldt-Universität zu Berlin, Berlin, Germany; 2 Epilepsy-Center Berlin-Brandenburg, Institute for Diagnostics of Epilepsy, Berlin, Germany

**Keywords:** Antiseizure medication, Prognosis, Stroke, Quality of life

## Abstract

**Background:**

In a globally ageing population, epilepsy in older adults has gained increased clinical relevance. This includes both individuals who have grown old with epilepsy and those who develop epilepsy later in life. Whether seizure outcomes and treatment patterns differ between these groups remains insufficiently characterised.

**Methods:**

In this retrospective study, we included patients aged ≥ 65 years treated at three outpatient epilepsy clinics in Berlin between 2010 and 2025. Patients were categorised according to age at epilepsy onset. Early-onset persistent epilepsy (EOPE) was defined as onset < 40 years, and late-onset epilepsy (LOE) as onset ≥ 65 years. The primary endpoint was seizure freedom during the last 12 months. Secondary endpoints included markers of antiseizure medication burden, adverse effects and quality of life.

**Results:**

A total of 243 patients were included (114 EOPE, 129 LOE). Seizure freedom was less frequent in EOPE compared to LOE (50.0% vs. 69.0%, *p* = 0.003). Patients with EOPE had a significantly higher drug load (median defined daily dose 1.00 vs. 0.67; *p* < 0.001). EOPE was independently associated with, among other variables, lower odds of seizure freedom (adjusted odds ratio (adjOR) 0.34, 95%CI 0.16–0.71) and more frequent use of first- or second-generation ASM (adjOR 5.99, 95%CI 2.47–14.54). The latter finding remained robust in a sensitivity analysis restricted to seizure-free patients.

**Conclusions:**

In older adults, EOPE and LOE are associated with differing clinical characteristics. Patients with EOPE show lower seizure freedom and higher treatment burden as compared to LOE. In seizure-free patients with long-standing epilepsy, ASM simplification may be considered.

**Supplementary Information:**

The online version contains supplementary material available at 10.1007/s00415-026-13916-9.

## Introduction

Epilepsy is a long-standing disorder and often accompanies patients for their entire lives. Its incidence follows a U-shaped distribution, with the highest rates in early childhood as well as in advanced age [1]. In the context of ongoing demographic change with a globally ageing population, the investigation of epilepsy in older adults has gained considerable clinical relevance.

Generally, there is no universally accepted definition of “epilepsy in the elderly”. In most studies, the term refers to epilepsy in individuals aged at least 60 or 65 years, reflecting commonly used age thresholds for defining older adulthood in medical research [2–5]. However, older adults with epilepsy do not constitute a homogeneous patient population. Two main groups can be distinguished: Individuals who have grown old with epilepsy following an early disease onset (“early-onset persistent epilepsy, EOPE”), and those who develop epilepsy in later life (“late-onset epilepsy, LOE”). The latter has been well studied with regard to incidence, aetiology, and clinical course; it is predominantly structural in nature, most commonly related to cerebrovascular and neurodegenerative causes [6–9]. In contrast, patients with early-onset epilepsy persisting into old age represent a population that has been far less systematically investigated and exhibits a more heterogeneous aetiological spectrum. In this group the underlying cause is most frequently classified as unknown, while identified aetiologies include non-lesional causes (such as genetic alterations) as well as lesions other than cerebrovascular disease and neurodegeneration [10, 11].

A recent Chinese cohort study demonstrated that elderly patients with LOE were more likely to achieve seizure freedom and required fewer antiseizure medications (ASM) than those with EOPE [12]. Older adults with epilepsy represent a particularly vulnerable patient group, as age-related factors, such as reduced drug metabolism, increased susceptibility to adverse effects, altered medication tolerability, and a higher risk of drug–drug interactions substantially influence ASM management [13–16]. Consequently, data from well-characterised cohorts, particularly addressing treatment burden and patient-reported outcomes in these distinct groups, are needed.

The aim of the present study was, therefore, to systematically assess and compare older patients with EOPE and those with LOE with regard to pharmacotherapy, response to antiseizure medication, and adverse effects to derive potential implications for more individualised and optimised therapy strategies in older age.

## Materials and methods

### Data source and patients

This retrospective study included consecutive patients from the three epilepsy outpatient clinics of the Department of Neurology at Charité–Universitätsmedizin Berlin between January 2010 and September 2025. These clinics are accessible to all epilepsy patients and do not specialise explicitly on difficult-to-treat cases. They use a shared electronic database and routinely assess adverse effects using the Liverpool Adverse Events Profile (LAEP), as well as self-reported quality of life and overall health status. Patients were eligible if they were ≥ 65 years old at their most recent visit, had a definite diagnosis of epilepsy according to the 2014 International League Against Epilepsy criteria [17] and had been followed in the outpatient setting for at least 1 year. Exclusion criteria were an uncertain epilepsy diagnosis, insufficient documentation, and a history of epilepsy surgery, as prior surgical treatment may substantially alter seizure outcomes and antiseizure medication requirements.

**Fig. 1:**
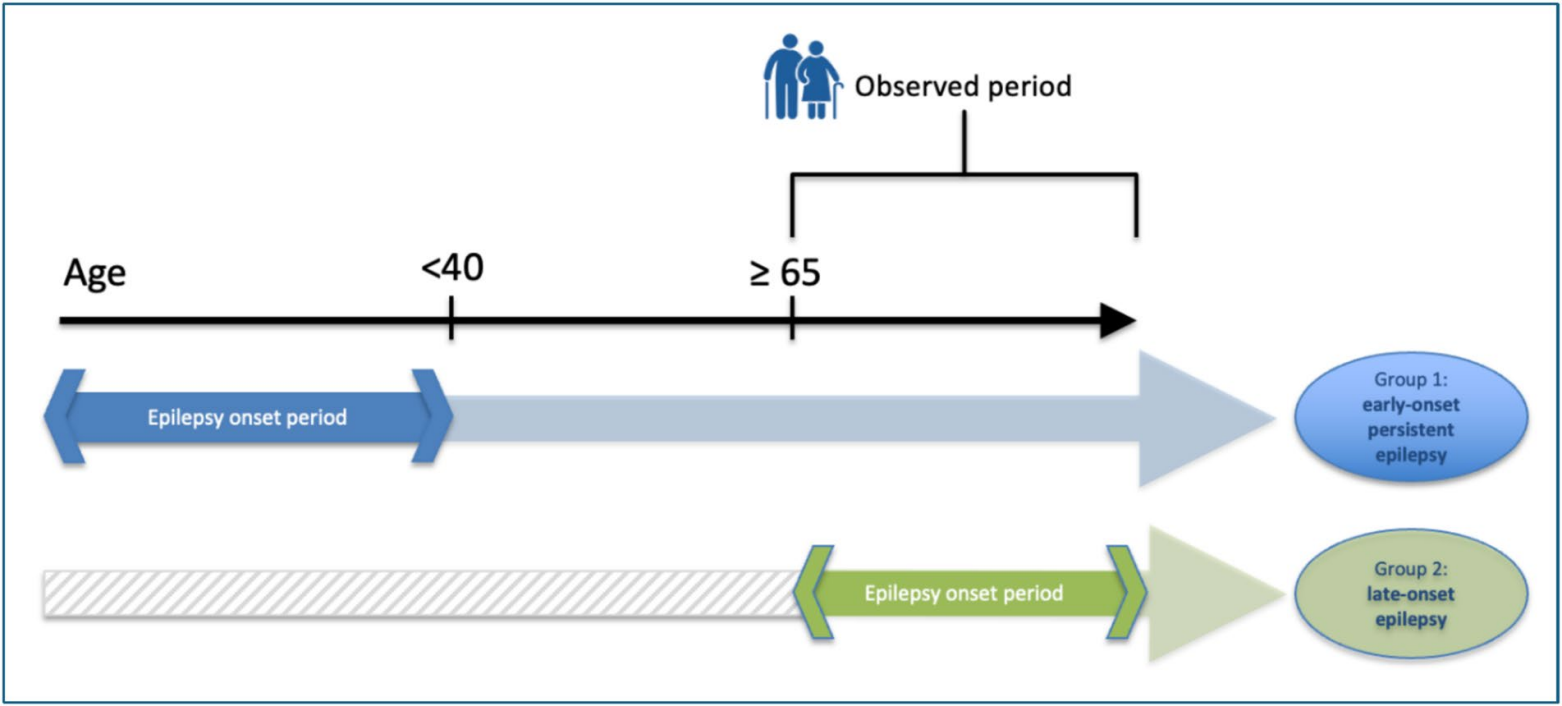
This figure illustrates the two patient groups analysed in this study. Patients with EOPE had their seizure onset period before the age of 40. Patients with LOE had their seizure onset period at ≥ 65 years. Patient outcomes were observed at ≥ 65 years for both patient groups

We categorised patients into two groups according to age at epilepsy onset (see Fig. 1).

- Patients ≥ 65 years old and diagnosed with epilepsy before the age of 40 years were classified into the early-onset persistent epilepsy group (EOPE). The term “early-onset” was chosen to clearly distinguish this group from late-onset epilepsy; however, the age threshold of 40 years was defined pragmatically for group differentiation and does not imply exclusively childhood-onset epilepsy.
- Patients ≥ 65 years old who received their first epilepsy diagnosis at ≥ 65 years were assigned to the late-onset epilepsy group (LOE).

For each included patient, the following variables were extracted from the database: age at last visit, age at first seizure, sex, duration of epilepsy, type of epilepsy (focal/generalised/unknown), history of generalised or bilateral tonic–clonic seizures, and seizure occurrence (generalised/bilateral tonic–clonic or focal) within the past 12 months. Regarding ASM therapy, the current regimen, including agent and dosage, and the presence of drug resistance as defined by the ILAE were recorded [18].

For each patient, the drug load at the last visit was calculated according to the 2020 Anatomical Therapeutic Chemical/Defined Daily Dose (ATC/DDD) Index of the World Health Organisation Centre for Drug Statistics Methodology, using the formula ‘individual ASM dosage per day divided by defined daily dose (DDD)’ [19]. For patients receiving multiple ASMs, the ratios were summed.

In addition, prescribed ASMs were classified as first-, second-, or third-generation agents according to the commonly used classification based on the year of regulatory approval [20]. First-generation ASM comprise compounds approved before 1958, such as ethosuximide, phenobarbital, and phenytoin. Second-generation ASM were introduced between 1960 and 1975, and include drugs, such as carbamazepine and valproate. The era of third-generation ASM began in the late 1980s and includes agents, such as lacosamide, lamotrigine, and levetiracetam.

Adverse effects of ASM were assessed with the Liverpool Adverse Events Profile (LAEP) which is routinely filled out by all patients treated in the Charité outpatient clinics. The LAEP is a self-administered questionnaire comprising 19 items that assess the frequency of commonly reported adverse effects experienced over the preceding 4 weeks. Responses are recorded on a four-point Likert scale ranging from 1 (“never”) to 4 (“often or always”). The summed score, therefore, varies between 19 and 76, with values of 45 or higher commonly interpreted as indicating a high burden of adverse effects [21, 22]. In addition, self-reported quality of life and overall health status were captured at each visit based on the documented results of two separate 11-point numerical rating scales. Both scales ranged from 0 (“worst possible”) to 10 (“best possible”) and referred to the 4 weeks preceding the respective visit.

The local ethics committee approved the study (EA2/181/20). Due to the retrospective nature of the study, the requirement for written informed consent was waived.

### Primary and secondary outcomes

Our primary endpoint was seizure freedom during the last 12 months prior to the last visit in our outpatient clinics. Our secondary endpoints were the presence of generalised/bilateral tonic–clonic seizures within the last 12 months and the individual ASM burden at the last documented visit. Further secondary endpoints were ASM adverse effects as assessed using the LAEP score at the most recent visit as well as self-reported quality of life and overall health status.

### Statistical analysis

The study population was compared between two groups: (1) patients with EOPE and (2) patients with LOE.

Data were checked for normal distribution using the Kolmogorov–Smirnov test. Continuous variables were summarised as the median and interquartile range (IQR), while categorical variables were presented as absolute numbers and relative frequencies. As none of the continuous variables followed a normal distribution, group comparisons of continuous variables were performed using the Mann–Whitney U test. Categorical variables were compared using the chi-square test. For contingency tables larger than 2 × 2 with small expected cell counts, the Fisher–Freeman–Halton exact test was used. The level of statistical significance was set at *p* < 0.05 (two-sided).

Multivariable logistic regression analyses were performed to identify clinical variables independently associated with EOPE. Three prespecified hierarchical models were constructed based on clinical reasoning.

- Model 1 included seizure freedom and demographic variables (sex and age at last outpatient visit).
- Model 2 additionally incorporated epilepsy type (focal vs. non-focal epilepsy).
- Model 3 further expanded the model to address antiseizure medication exposure and treatment intensity, including the number of ASM currently taken and the use of first or second-generation ASM.

All covariates were selected a priori based on clinical relevance and entered simultaneously into the models. Results are reported as odds ratios (ORs) with 95% confidence intervals (CIs).

In addition, a sensitivity analysis was conducted, restricting the analysis to patients who had been seizure-free during the last 12 months. The aim of this sensitivity analysis was to assess whether observed differences between the groups were independent of current seizure activity and thus not primarily attributable to ongoing seizures.

Statistical analyses were performed using IBM SPSS Statistics (version 30).

## Results

### Study population

**Fig. 2:**
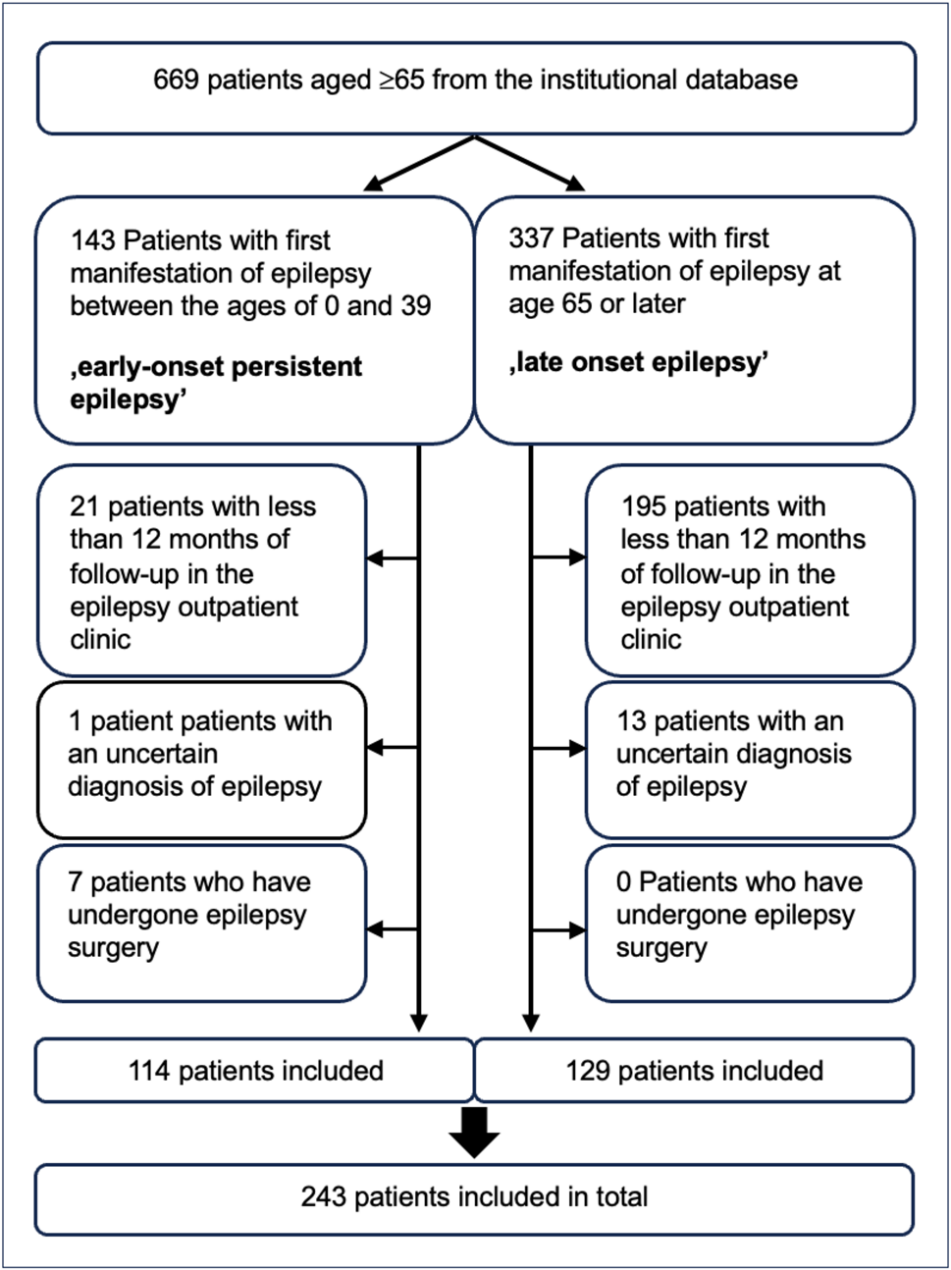
This shows patient selection and reasons for exclusion among the EOPE and LOE groups

From the institutional database, 669 patients aged 65 years or older were initially identified. Of these, 143 patients had epilepsy onset before the age of 40 years (i.e., early-onset persistent epilepsy) and 337 patients had epilepsy onset at 65 years of age or later (i.e., late-onset epilepsy). After detailed data review and application of the predefined exclusion criteria, a total of 243 patients were included in this study. Ultimately, 114 patients were assigned to the EOPE group and 129 patients to the LOE group. The patient selection process is illustrated in Fig. 2.

Patient characteristics are summarised in Table 1. Fifty-one percent of patients were female. The median age at the last outpatient visit was 76 years (interquartile range [IQR] 71–81 years).

**Table 1:** Comparison of early-onset persistent vs. late-onset epilepsy

Variable	All patients	Group 1 Early-onset persistent epilepsy (EOPE) *n* = 114	Group 2 Late-onset epilepsy (LOE) *n* = 129	*p* value
Age at last visit, median years (IQR)	76 (71–81)	72 (69–77)	78 (74–83)	**p < 0.001**^**b**^
Age at onset of epilepsy, median years (IQR)	65 (16.5–72)	16 (10–23)	72 (69–76)	**p < 0.001**^**b**^
Duration of epilepsy, median years (IQR)	14 (5–54.5)	55 (50–62)	5 (3–8)	**p < 0.001**^**b**^
Female sex, *n* (%)	125 (51.4%)	71 (62.3%)	54 (41.9%)	**p = 0.001**^**a**^
Type of epilepsy, *n* (%)				**p < 0.001**^**c**^
Focal, n (%)	207 (85.2%)	82 (71.9%)	125 (96.9%)	
Generalised, *n* (%)	24 (9.9%)	24 (21.1%)	0 (0%)	
Focal and generalised, *n* (%)	2 (0.8%)	2 (1.8%)	0 (0%)	
Unknown, *n* (%)	10 (4.1%)	6 (5.3%)	4 (3.1%)	
Aetiology of epilepsy				**p < 0.001**^**c**^
Structural, *n* (%)	141 (58.0%)	44 (38.6%)	97 (75.2%)	
Genetic, *n* (%)	23 (9.5%)	23 (20.2%)	0 (0%)	
Structural and genetic, *n* (%)	1 (0.4%)	1 (0.9%)	0 (0%)	
Immune, *n* (%)	3 (1.2%)	0 (0%)	3 (2.3%)	
Infectious, *n* (%)	1 (0.4%)	1 (0.9%)	0 (0%)	
Unknown, *n* (%)	74 (30.5%)	45 (39.5%)	29 (22.5%)	
Seizure-free last 12 months, *n* (%)	146 (60.1%)	57 (50.0%)	89 (69.0%)	**p = 0.003**^**a**^
B/GTCS last 12 months, *n* (%)	24 (9.9%)	18 (15.8%)	6 (4.7%)	**p = 0.004**^**a**^
B/GTCS ever, *n* (%)	172 (70.8%)	102 (89.5%)	70 (54.3%)	**p < 0.001**^**a**^
Antiseizure medication				
Number of current ASM, median (IQR)	1 (1–2)	1 (1–2)	1 (1–1)	**p = 0.003**^**b**^
ATC/DDD, median (IQR)	0.83 (0.5–1.48)	1 (0.67–1.70)	0.67 (0.38–1.00)	**p < 0.001**^**b**^
1st generation ASM, *n* (%)	15 (6.2%)	15 (13.2%)	0 (0%)	**p < 0.001**^**a**^
2nd generation ASM, *n* (%)	48 (19.8%)	38 (33.3%)	10 (7.8%)	**p < 0.001**^**a**^
3rd generation ASM, *n* (%)	185 (76.1%)	75 (65.8%)	110 (85.3%)	**p < 0.001**^**a**^
No ASM	17 (7.0%)	3 (2.6%)	14 (10.9%)	**p < 0.001**^**a**^
Valproate, *n* (%)	26 (10.7%)	20 (17.5%)	6 (4.7%)	**p = 0.001**^**a**^
Levetiracetam, n (%)	92 (37.9%)	32 (28.1%)	60 (46.5%)	**p = 0.003**^**a**^
Lamotrigine, *n* (%)	69 (28.4%)	36 (31.6%)	33 (25.6%)	p = 0.301^a^
Drug resistance, *n* (%)	52 (21.4%)	46 (40.4%)	6 (4.7%)	**p < 0.001**^**a**^
LAEP sum score (IQR)	35 (27–43)	35 (27–43)	36 (27–43)	p = 0.726^b^
Self-rated health scale, median (IQR)	5 (4–8)	5 (4–7)	5 (4–8)	p = 0.624^b^
Quality of life score, median (IQR)	6 (4–8)	5 (4–8)	6 (4–8)	p = 0.188^b^

Bold indicates statistically significant

*IQR* interquartile range; *n* number, *B/GTCS* bilateral or generalised tonic–clonic seizure, *ASM* antiseizure medication, *DDD* defined daily dose, *LAEP* Liverpool Adverse Events Profile

^a^Chi-squared test

^b^Mann–Whitney *U*-test

^c^Freeman–Halton exact test

At the time of data collection, 160 patients (65.8%) were treated with ASM monotherapy, 66 patients (27.1%) received polytherapy, and 17 (7.0%) were not receiving any ASM. The most frequently prescribed ASM was levetiracetam (37.9%), followed by lamotrigine (28.4%).

At the last outpatient visit, 60.1% of the entire cohort had been seizure-free for at least the last 12 months. Drug-resistant epilepsy was present in 52 patients (21.4%).

### Comparison of patients with EOPE and LOE

**Fig. 3:**
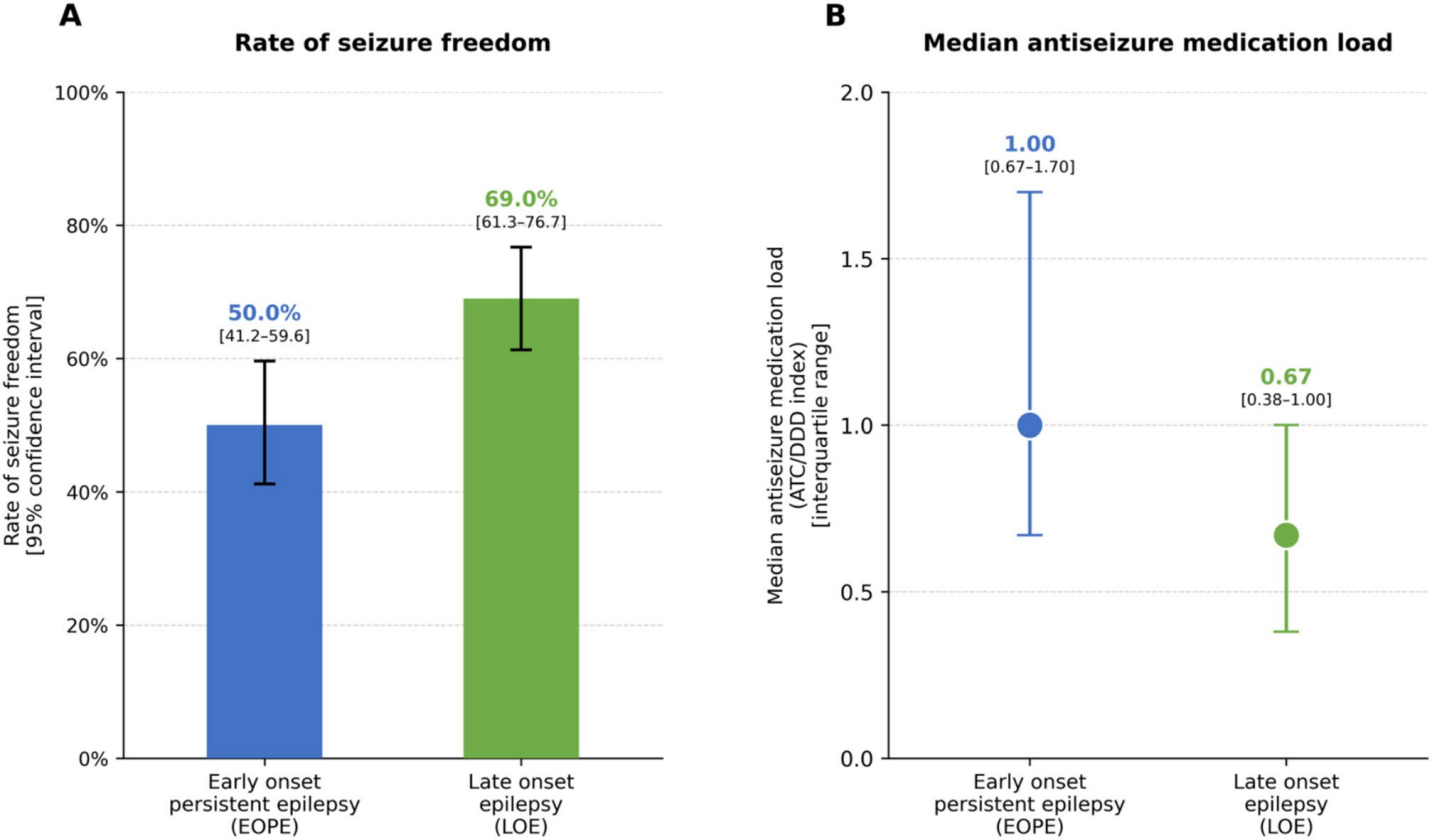
Comparison of seizure freedom rates and ASM loads between the EOPE and LOE groups. **A** Bars indicate the proportion of patients achieving seizure freedom for each group. Error bars represent 95% confidence intervals. **B** Dots represent the median antiseizure medication load; vertical lines denote the interquartile range. ASM load was calculated in accordance with the 2020 WHO Centre for Drug Statistics Methodology ATC/DDD Index, using the following formula: the individual antiseizure medication dosage per day divided by the defined daily dose

The rate of seizure-freedom was 50.0% (95% CI 41.2–59.6) in the EOPE group and 69.0% (95% CI 61.3–76.7) in the LOE group (*p* = 0.003). Patients with EOPE showed a significantly higher antiseizure medication (ASM) burden. This was reflected by a significantly higher ATC/DDD index (median 1.00 vs. 0.67; *p* < 0.001). The rate of seizure-freedom and ATC/DDD index for both patients with EOPE and LOE are illustrated in Fig. 3.

Drug-resistant epilepsy was significantly more common in the EOPE group as compared to the LOE group (40.4% vs. 4.7%; *p* < 0.001). Table 1 contrasts clinical variables in patients with EOPE and LOE.

The results of the multivariable logistic regression analyses are presented in Table 2. In the fully adjusted model (model 3), EOPE was independently associated with lower odds of seizure freedom (OR 0.34, 95% CI 0.16–0.71) and with a more frequent use of first- or second-generation ASM (OR 5.99, 95% CI 2.47–14.54). EOPE was not independently associated with the number of currently used ASM (OR 1.2, 95% CI 0.7–2.04).

**Table 2:** Multivariable logistic regression models for early-onset persistent epilepsy

Variable	Model 1 OR (95% CI)	Model 2 OR (95% CI)	Model 3 OR (95% CI)
Seizure-free last 12 months	0.39 (0.22–0.71)	0.33 (0.17–0.62)	0.34 (0.16–0.71)
Age at last visit (per year)	0.86 (0.82–0.90)	0.85 (0.81–0.90)	0.85 (0.80–0.90)
Female sex	2.09 (1.18–3.72)	2.14 (1.16–3.96)	2.21 (1.14–4.28)
Focal epilepsy* (vs. non-focal)	–	0.07 (0.02–0.22)	0.10 (0.03–0.38)
Number of ASM currently taken	–	–	1.20 (0.70–2.04)
Use of first- or second-generation ASM	–	–	5.99 (2.47–14.54)

*ASM* antiseizure medication

*Epilepsy type was entered into the multivariable model as a binary variable (focal vs. non-focal epilepsy) to avoid model instability due to quasi-complete separation

In our analysis, there was no difference between patients with EOPE and LOE with regards to adverse effects expressed as median LAEP scores (35 (27–43) vs. 36 (27–43), *p* = 0.726), self-rated health score (5 (4–7) vs. 5 (4–8), *p* = 0.624), and quality of life score (5 (4–8) vs. 6 (4–8), *p* = 0.188).

### Sensitivity analysis on seizure-free patients

The results of the sensitivity analysis, in which only patients with seizure freedom for at least the terminal 12 months were included, are listed in supplementary Table S1 (univariable analysis) and S2 (multivariable analysis).

In the multivariable analysis, EOPE remained strongly associated with the use of first- or second-generation ASM (OR 31.38, 95% CI 6.72–146.56). A significant association also remained between EOPE and younger age at last index visit (OR 0.79, 95% CI 0.71–0.88) as well as lower odds of focal epilepsy (OR 0.13, 95% CI 0.02–0.76). As in our primary analysis, there was still no significant association between EOPE and the current number of ASMs (OR 0.72, 95% CI 0.26–2.01).

## Discussion

The key finding of this study is that epilepsy in older age does not represent a homogeneous entity: patients who have grown old with epilepsy differ clinically from those who develop epilepsy later in life. In our cohort, patients with early-onset persistent epilepsy showed lower rates of seizure freedom, more frequently a history of bilateral or generalised tonic–clonic seizures, a higher ASM burden, and significantly higher rates of drug resistance compared to patients with late-onset epilepsy.

Traditionally, epilepsy in older age has been considered a condition with relatively favourable treatment response and high rates of seizure freedom [10, 23, 24]. However, treatment response in older adults with epilepsy has rarely been examined with respect to age at disease onset. A recent retrospective cohort study from China demonstrated a lower rate of seizure freedom in patients whose epilepsy persisted into old age as compared to late-onset epilepsy [12]. Notably, these findings were derived from univariable analyses and were not adjusted for demographic, aetiological, or treatment-related factors.

Importantly, these observations as well as our current findings must be interpreted in the context of potential referral and follow-up bias: patients with EOPE who continue to be treated in a tertiary epilepsy outpatient centre at an older age might be likely to represent a selected subgroup of patients with more difficult-to-treat epilepsy. This could result in an overrepresentation of more complex cases with drug resistance within the EOPE group. Future studies using national or population-based datasets may help to validate our findings in cohorts less prone to referral and follow-up bias and thereby improve generalisability.

To mitigate the impact of this potential bias, a differentiated statistical approach was required:

First, by applying a multivariable regression analysis, we demonstrate that the observed differences in seizure freedom between EOPE and LOE remain significant after adjustment for clinically relevant covariates including age, sex, epilepsy type, and ASM burden.

Adjustment for age was particularly important, as the median age at the time of observation differed between the two groups, primarily for methodological reasons, with patients in the LOE group, as expected, being older. By adjusting for age, we were able to disentangle the effect of age at epilepsy onset from differences in age distribution between the groups.

In addition, adjustment for epilepsy type was essential, as the EOPE group naturally had a higher proportion of non-focal epilepsy while the late-onset epilepsy group consisted almost exclusively of focal epilepsies of structural origin. While studies in the general epilepsy population have reported lower seizure freedom rates for focal epilepsies (48–62%) [25–27] compared to idiopathic generalised epilepsies (68–85%) [25, 28–30], these data are largely derived from age-independent cohorts and do not specifically address older patients. Interestingly, in our multivariable analysis, differences in seizure freedom remained significant after adjustment for epilepsy type. Thus, our findings indicate that the less favourable seizure outcome observed in EOPE is independent of epilepsy type. This suggests that disease chronicity and long-term treatment trajectories contribute to differences in seizure control in older age beyond epilepsy type alone. Nevertheless, differences in underlying epilepsy aetiologies between EOPE and LOE may also substantially contribute to the observed differences in seizure outcomes and treatment burden. Future studies with larger cohorts and aetiology-focused classifications are needed to further disentangle the respective contributions of age at onset, disease duration, and epilepsy cause. Regarding focal epilepsies, late-onset epilepsy is mainly caused by cerebrovascular and neurodegenerative diseases [3, 6, 8, 9]. Focal epilepsies caused by ischaemic stroke have been shown to exhibit the highest rates of seizure freedom (> 70%) with the lowest ASM burden as well as the lowest rate of breakthrough seizures after seizure freedom for at least 1 year in comparison to other epilepsy aetiologies [27, 31]. In contrast, focal epilepsies due to other aetiologies, such as hippocampal sclerosis or cerebrovascular malformations, typically manifest earlier in life, which may partly explain the inferior prognosis of EOPE [32].

Recent data further highlight the prognostic relevance of aetiology within late-onset epilepsy itself: a retrospective cohort study demonstrated that late-onset epilepsy of unknown aetiology was significantly more treatment-responsive than lesional late-onset epilepsy [33]. These findings suggest that not only age at onset, but also the underlying cause of epilepsy substantially influences seizure outcomes in older adults.

Another important finding that has not been studied previously is that patients with EOPE were exposed to a substantially higher ASM burden. This was reflected by a higher drug load and more frequent use of first- or second-generation ASM. Older patients represent a particularly vulnerable population with respect to ASM therapy. Age-related factors, such as reduced drug metabolism, altered tolerability, and increased susceptibility to adverse effects must be considered [34, 35]. A multicentre cross-sectional study has shown that adverse effects increase with the number of ASMs, particularly in polytherapy [36]. Consequently, ASM monotherapy is generally recommended as first-line treatment in older patients, irrespective of age at epilepsy onset [37]. In our cohort, two thirds of patients received monotherapy, and three in four patients were treated with third-generation ASM. Nevertheless, patients with early-onset persistent epilepsy (including those that were seizure-free) continued to receive first- or second-generation ASM more frequently. Many of these patients have been treated for decades with older ASMs, which are known to be associated with higher rates of fatigue, cognitive impairment, and other adverse effects compared with newer agents [38].

Against this background, it should be discussed whether older patients, particularly those who are seizure-free and who have grown old with their epilepsy diagnosis, might benefit from a simplification of their ASM regimen, including a dose reduction, a switch to monotherapy or to a better tolerated third-generation ASM. Decisions regarding ASM withdrawal or dose reduction in sustained seizure freedom are complex and highly individualised. Previous studies have shown that more than 80% of patients choose to continue their ASM despite long-term seizure freedom [39, 40]. Further insights into decision-making processes in older adults may help clarify barriers to therapy modification in this population.

An additional argument supporting the clinical consideration of a dose reduction or treatment simplification arises from the dissociation we observed between seizure freedom and ASM burden in our cohort. As illustrated in Fig. 3, patients with early-onset persistent epilepsy were less likely to be seizure-free despite a higher median drug load, whereas patients with late-onset epilepsy achieved higher rates of seizure freedom at lower treatment intensity. This inverse pattern suggests that, in older age, higher treatment intensity in early-onset persistent epilepsy does not necessarily translate into superior seizure control. At the same time, the increased ASM burden may reflect greater disease severity and long-standing drug resistance.

In light of all these considerations, an additional and particularly important aspect of our study is the sensitivity analysis restricted to seizure-free patients. By focusing exclusively on individuals without recent seizures in the last 12 months, we were able to examine whether differences in treatment burden were potentially driven by ongoing seizures or reflected more fundamental differences related to age at onset of epilepsy. Importantly, the key findings of the primary analysis remained largely robust in the sensitivity analysis restricted to seizure-free patients. Even among individuals who were seizure-free, early-onset persistent epilepsy was strongly associated with the use of first- or second-generation ASM. This finding remained statistically significant, even after adjusting for epilepsy type. Thus, our findings underscore that differences in treatment patterns between EOPE and LOE are not solely a consequence of insufficient seizure control, but are strongly influenced by long-standing epilepsy and ASM treatment history.

Despite the higher ASM burden in the group with EOPE, no significant differences were found in terms of adverse effects, quality of life, or subjective health as assessed by standardised scores. This suggests that a higher ASM burden in our patient population is not necessarily associated with a poorer subjective perception. This finding was somewhat surprising, as higher ASM burden has generally been associated with reduced quality of life [41]. One possible explanation for the comparable quality of life observed in patients with early-onset persistent epilepsy despite higher ASM load is that older individuals with long-standing epilepsy may adapt to chronic treatment-related symptoms. In addition, age-related functional limitations and health complaints common to both groups may outweigh or mask more subtle ASM-related adverse effects, thereby attenuating perceived differences in quality of life. However, these results also must be interpreted in light of the limited sensitivity of the instruments used, in particular, the simple rating scales for health and quality of life.

This study has several limitations. The most relevant potential limitation, namely referral and follow-up bias in the EOPE group, has already been discussed above. In addition, although patients were recruited from three outpatient clinics with a heterogeneous patient population, all sites belong to a single tertiary care centre. The study should, therefore, be considered monocentric, which may limit generalisability. The use of distinct age-at-onset cut-offs to define EOPE and LOE (i.e., onset < 40 years vs. onset ≥ 65 years) was a deliberate methodological choice aimed at ensuring clear group separation. However, this approach may not capture the full spectrum of epilepsy trajectories in older adults. Finally, the retrospective design precludes causal inference and does not fully eliminate the possibility of residual confounding, despite multivariable adjustment.

## Conclusions

In summary, our results support the view that early-onset persistent epilepsy and late-onset epilepsy in older adults differ substantially with regard to seizure outcomes and ASM treatment burden. Patients with EOPE were less likely to achieve seizure freedom, yet they carried a substantially higher ASM burden. This increased treatment burden was reflected by a higher cumulative drug load and more frequent use of first- or second-generation compounds. Importantly, this increased treatment burden persisted even among seizure-free individuals. These findings suggest that long-term epilepsy and treatment histories strongly influence therapeutic complexity in older age. In seizure-free patients with early-onset persistent epilepsy, the potential for simplification of ASM regimens, such as dose reduction, monotherapy, or switching to better tolerated modern agents, may, therefore, warrant consideration.

## Supplementary Information

Below is the link to the electronic supplementary material.

Supplementary file1 (DOCX 27 KB)

## Data Availability

The data that support the findings of this study are available from the corresponding author upon reasonable request.
