# Supplementary material for: Epilepsy in older adults: a comparison of early-onset persistent and late-onset epilepsy
Source: J Neurol. 2026 Jun 9;273(7):383. doi: 10.1007/s00415-026-13916-9 (PMC13249777; doi:10.1007/s00415-026-13916-9)
Supplement: Supplementary file 1 — Supplementary file1 (DOCX 27 KB) [file 415_2026_13916_MOESM1_ESM.docx]

**Epilepsy in older adults: a comparison of early-onset persistent and late-onset epilepsy**

Nicoletta Dörr^1^, Thea Hüsing^1^, Hanna Kleespies^1^, Martin Holtkamp^1,2^, Jakob I Doerrfuss^1^

Corresponding Author: Nicoletta Dörr, Department of Neurology, Charité́ – Universitätsmedizin Berlin Campus Benjamin Franklin, Email: nicoletta.doerr@charite.de

**Supplementary material**

| **Table S1: Comparison of early-onset persistent vs. late-onset epilepsy - Sensitivity analysis on seizure-free patients** | | | |
| --- | --- | --- | --- |
| **Variable** | **Group 1  Early-onset persistent epilepsy n=57** | **Group 2  Late onset epilepsy  n=89** | **P value** |
| **Age at last visit, median years (IQR)** | 71 (69-75) | 78 (74-83) | **p<0.001^b^** |
| **Age at onset of epilepsy, median years (IQR)** | 17 (12-23) | 72 (69-77) | **p<0.001^b^** |
| **Duration of epilepsy, median years (IQR)** | 54 (49-59) | 5 (3-7) | **p<0.001^b^** |
| **Female sex, n (%)** | 35 (61.4%) | 34 (38.2%) | **p=0.006^a^** |
| **Type of epilepsy, n (%)** |  |  | **p<0.001^c^** |
| **focal, n (%)** | 40 (70.2%) | 85 (95.5%) |  |
| **generalised, n (%)** | 15 (26.3%) | 0 (0%) |  |
| **unknown, n (%)** | 2 (3.5%) | 4 (4.5%) |  |
| **B/GTCS ever, n (%)** | 50 (87.7%) | 47 (52.8%) | **p<0.001^a^** |
| **Antiseizure medication** |  |  |  |
| **Number of current ASM, median (IQR)** | 1 (1-1) | 1 (1-1) | **p=0.041^b^** |
| **ATC/DDD** | 0.67 (0.4-1.3) | 0.67 (0.33-1) | p=0.074^b^ |
| **1^st^ generation ASM, n (%)** | 10 (17.5%) | 0 (0%) | **p<0.001^a^** |
| **2^nd^ generation ASM, n (%)** | 24 (42.1%) | 4 (4.5%) | **p<0.001^a^** |
| **3^rd^ generation ASM, n (%)** | 27 (47.4%) | 76 (85.4%) | **p<0.001^a^** |
| **no ASM, n (%)** | 3 (5.3%) | 11 (12.4%) | p =0.155**^a^** |
| **Valproate, n (%)** | 15 (26.3%) | 3 (3.4%) | **p<0.001^a^** |
| **Levetiracetam, n (%)** | 14 (24.6%) | 39 (43.8%) | **p=0.018^a^** |
| **Lamotrigine, n (%)** | 8 (14%) | 23 (25.8%) | p=0.089^a^ |
| **Drug resistance, n (%)** | 9 (15.8%) | 0 (0%) | **p<0.001^a^** |
| **LAEP sum score (IQR)** | 33 (24-40) | 35 (27-42) | p=0.243^b^ |
| **Self-rated health scale, median (IQR)** | 7 (5-8) | 6 (4-8) | p=0.241^b^ |
| **Quality of life score, median (IQR)** | 7 (5-9) | 6 (4-8) | p=0.239^b^ |

*Note:* Bold indicates statistically significant.

IQR=interquartile range; n=number, B/GTCS=bilateral or generalised tonic-clonic seizure, ASM=antiseizure medication, DDD=defined daily dose, LAEP = Liverpool Adverse Events Profile

aChi-squared test

bMann-Whitney *U*-test

cFreeman-Halton exact test

| **Table S2 - Multivariable logistic regression models for early-onset persistent epilepsy – Sensitivity analysis on seizure-free patients** | | | |
| --- | --- | --- | --- |
| **Variable** | **Model S1 OR (95% CI)** | **Model S2 OR (95% CI)** | **Model S3 OR (95% CI)** |
| Age at last visit (per year) | 0.81 (0.75–0.88) | 0.80 (0.73–0.87) | 0.79 (0.71–0.88) |
| Female sex | 1.86 (0.84–4.08) | 2.17 (0.92–5.10) | 2.65 (0.95–7.44) |
| Focal epilepsy* (vs. non-focal) | — | 0.07 (0.02–0.28) | 0.13 (0.02–0.76) |
| Number of ASM currently taken | — | — | 0.72 (0.26–2.01) |
| Use of first- or second-generation ASM | — | — | 31.38 (6.72–146.56) |

* Epilepsy type was entered into the multivariable model as a binary variable (focal vs. non-focal epilepsy) to avoid model instability due to quasi-complete separation.

ASM=antiseizure medication
